# Promoting Regional Health Cooperation: The South Asian Public Health Forum

**DOI:** 10.1371/journal.pmed.0030108

**Published:** 2006-05-09

**Authors:** Rana Jawad Asghar

## Abstract

One of the ways in which health professionals can help to improve the poor state of public health in South Asia is through regional cooperation and collaboration.

The seven countries of South Asia (Bangladesh, Bhutan, India, Maldives, Nepal, Pakistan, and Sri Lanka) have a collective population of nearly 1.4 billion people [ 1]. This region has a long history of hostility and mistrust among its nations, and heavy governmental spending on defense has diverted resources away from investing in health and education [ 2–5]. Forty percent of people in South Asia live on less than one dollar per day [ 6], half of all children below the age of five years are malnourished [ 7], and the region's 45% illiteracy rate is the highest in the world [ 8].

One of the ways in which health professionals can help to improve this poor state of public health in South Asia is through regional cooperation and collaboration. This spirit of cooperation was the driving force behind the formation in 1999 of the South Asian Public Health Forum (SAPHF; http://www.saphf.org). This independent voluntary organization aims to improve communication and interaction between public health professionals working in South Asia or those working outside the region who have an interest in South Asia. It also disseminates public health information to the broader public. In this article, I discuss why the forum was launched, how it operates, the impact that it has had, and the challenges we face.

## The Need for Regional Cooperation

Over the last two decades, efforts have been made to bring the countries of South Asia together. In 1985, the South Asian Association for Regional Cooperation (SAARC) was established at a meeting of heads of states ( http://www.saarc-sec.org). The commitment to cooperate came from the top, which was both the strongest asset of SAARC and also its weakest link. As an intergovernmental organization, SAARC was held hostage to the political rivalries of different governments. Except for brief and intermittent periods, SAARC was unable to pave the way to meaningful regional cooperation. [Fig pmed-0030108-g001]

**Figure pmed-0030108-g001:**
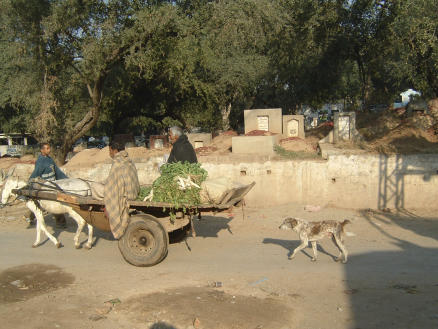
Forty percent of people in South Asia live on less than one dollar per day (Photo: Rana Jawad Asghar)

The two biggest countries of the region—India and Pakistan—were largely responsible for these deadlocks. Hostility between these two countries reached its zenith in 1998, when both nations exploded their atomic bombs. By 1999 there was the real possibility of an open war, and over the following years, “the subcontinent and its military and political leadership … moved seamlessly from an obtuse nuclear capability and a doctrine of nuclear deterrence to the present state of nuclear weaponisation” [ 9].

There are many ways in which this hostility between South Asian countries may have contributed towards impeding health in the region in the 1980s and 1990s. For example, it probably impeded infectious disease control efforts—such diseases are rampant in the region, they do not respect national boundaries, and they require regional cooperation for their control. Infectious and parasitic diseases account for about 20% of the disability-adjusted life years in the region, and their recent rise was compounded by drug resistance, re-emergence of infections such as malaria and typhoid, and emergence of new infections such as dengue and HIV [ 10]. [Fig pmed-0030108-g002]

**Figure pmed-0030108-g002:**
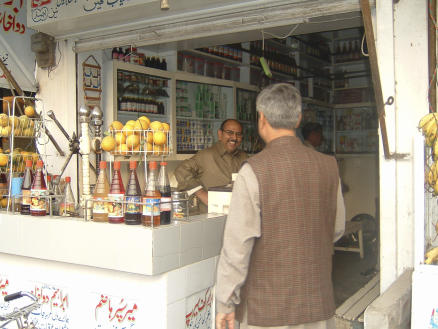
A spirit of cooperation will be crucial for development in South Asia (Photo: Rana Jawad Asghar)

SAPHF grew out of a belief among a group of health professionals—some of whom were distinguished public and international health experts—that it was crucially important to keep a channel of communication open between countries in the region, regardless of the hostilities between nations. Only an independent, nongovernmental body could have provided such a channel. Our objective was simple: to improve communication between public health professionals and encourage them to discuss and to share important health issues of the region quickly and without interference from governments. Our vision was that the sharing of information could lead to better health policies, which would eventually benefit the communities we serve.

## How the Forum Operates

SAPHF was keen to be financially independent, and its members volunteer their time freely. Its online presence was maintained through a free Web site–hosting service (there is now a nominal monthly fee), and its E-mail list (which is open to all) grew from its early beginnings in April 1999.

Our main activity is to collect crucial news and articles on public health in South Asia from a variety of different resources and then circulate them among our members. We prioritize emergency health issues, such as outbreaks, and we scan local newspapers and online resources such as ProMED-mail (a freely available global electronic reporting system for outbreaks of emerging infectious diseases, at http://www.promedmail.org). Over the years some important outbreaks have been simultaneously reported via SAPHF and ProMED-mail.

We also post clinical research articles or their abstracts from the South Asian region with links to the full text of original articles whenever possible. We have included discussions of health economics, but we have refrained from posting anything that is too overtly political—to prevent fueling any international tensions.

## The Forum's Impact

From very early on, our E-mail list became more popular among news agencies and newspapers than among public health professionals. We recently discovered that an important story in Pakistan's biggest newspaper was based on a posting on SAPHF. We have collected and circulated nearly 3,000 news items, articles, job postings, notification of outbreaks, and personal comments on public health among our members, all of which are archived and publicly available and which can be searched using a keyword.

Even though many postings were critical of one government or another, only on a handful of occasions has SAPHF been accused of any political bias. Considering the level of hostility between countries in our region, we feel vindicated in choosing our approach of focusing on health-related issues and avoiding highly explosive political issues.

Over the years, as health professionals from the region have started respecting and understanding the need for public health, our membership has increased and we now have nearly 700 professionals. These professionals are spread all over the world but have interest in South Asia for academic reasons, because of their ethnicity, or both.

SAPHF is still an independent and voluntary organization and has been recognized and appreciated by many organizations and institutions interested in the region. We are part of an initiative called Access to Information for Health Professionals in Developing Countries (see http://www.inasp.info/health/index.shtml), coordinated by the International Network for the Availability of Scientific Publications (INASP; http://www.inasp.info). A *BMJ* theme issue on health in South Asia (the theme issue is at http://bmj.bmjjournals.com/content/vol328/issue7443/) highlighted our work in its review of South Asian health Web sites [ 11].

## Next Steps

With no financial resources and no “official” employees, maintaining this forum has been difficult. The next challenge will come when we need to expand the SAPHF. Transforming a small, informal organization such as the SAPHF into a larger, more official project will be a big step, requiring formal commitment of time and resources.

## Abbreviations

SAARC: South Asian Association for Regional Cooperation
SAPHF: South Asian Public Health Forum
